# Pupil Dilation during Reward Anticipation Is Correlated to Depressive Symptom Load in Patients with Major Depressive Disorder

**DOI:** 10.3390/brainsci10120906

**Published:** 2020-11-25

**Authors:** Max Schneider, Immanuel G. Elbau, Teachawidd Nantawisarakul, Dorothee Pöhlchen, Tanja Brückl, BeCOME Working Group, Michael Czisch, Philipp G. Saemann, Michael D. Lee, Elisabeth B. Binder, Victor I. Spoormaker

**Affiliations:** 1 Department of Translational Research in Psychiatry, Max Planck Institute of Psychiatry, 80804 Munich, Germany; max_schneider@psych.mpg.de (M.S.); immanuel_elbau@psych.mpg.de (I.G.E.); nantawisarakul@psych.mpg.de (T.N.); dorothee_poehlchen@psych.mpg.de (D.P.); brueckl@psych.mpg.de (T.B.); become@psych.mpg.de (BeCOME Working Group); czisch@psych.mpg.de (M.C.); saemann@psych.mpg.de (P.G.S.); binder@psych.mpg.de (E.B.B.); 2 Department of Cognitive Sciences, University of California, Irvine, CA 92697-5100, USA; mdlee@uci.edu

**Keywords:** depression, arousal, reward, pupillometry, fMRI

## Abstract

Depression is a debilitating disorder with high prevalence and socioeconomic cost, but the brain-physiological processes that are altered during depressive states are not well understood. Here, we build on recent findings in macaques that indicate a direct causal relationship between pupil dilation and anterior cingulate cortex mediated arousal during anticipation of reward. We translated these findings to human subjects with concomitant pupillometry/fMRI in a sample of unmedicated participants diagnosed with major depression and healthy controls. We could show that the upregulation and maintenance of arousal in anticipation of reward was disrupted in patients in a symptom-load dependent manner. We could further show that the failure to maintain reward anticipatory arousal showed state-marker properties, as it tracked the load and impact of depressive symptoms independent of prior diagnosis status. Further, group differences of anticipatory arousal and continuous correlations with symptom load were not traceable only at the level of pupillometric responses, but were mirrored also at the neural level within salience network hubs. The upregulation and maintenance of arousal during reward anticipation is a novel translational and well-traceable process that could prove a promising gateway to a physiologically informed patient stratification and targeted interventions.

## 1. Introduction

Major depressive disorder (MDD) is a mental disorder with a high prevalence and is estimated to be the top contributor to nonfatal health loss globally [1]. Experimental work has revealed that disturbances in the positive valence domain—as assessed with reward tasks—may be central to depressive symptomatology [2,3,4,5]. The reward system comprises of various subprocesses that serve different functions within the anticipation, approach, and consumption of rewarding stimuli. Within the context of depression, much attention has been devoted to the processes of reward prediction and prediction error signaling. This followed the hypothesis that the common finding of anhedonia in depression mirrors deficits in predicting the value of an anticipated stimulus. Several studies have indeed observed group differences in the striatum during reward anticipation, when the task contained a learning component [6,7,8]. This was not the case in a nonlearning task: Rutledge et al. [9] showed that individuals with depression exhibit reward prediction and prediction error signals in the ventral striatum similar to controls. Based on this, it was proposed that evidence for attenuated prediction error signaling in depression could mirror downstream effects more closely related to aberrant behavior.

One such downstream subprocess is the upregulation and sustainment of arousal after reward prediction. Rudebeck et al. [10] used pupillometry to track sustained arousal to an expected reward in macaque monkeys, and revealed that a lesion in the subgenual anterior cingulate cortex (in a few cases extending to the dorsal anterior cingulate) impaired sustained arousal in a reward delay task. Anatomical evidence indicates direct functional connections from the dorsal anterior cingulate and medial prefrontal cortex to the locus coeruleus (the main noradrenergic output center in the brain) in macaque monkeys [11]; this provides an anatomical pathway through which reward-induced salience could lead to physiological arousal. In our previous pupillometry/functional magnetic resonance imaging (fMRI) study [12], we translated these findings to healthy subjects by employing a well validated reward anticipation task [13]. We observed that the change in pupil size (*pupil dilation*) during reward anticipation was associated with activity in the dorsal anterior cingulate and bilateral insula, i.e., the salience network. This upregulation of arousal during reward anticipation likely facilitates reward approaching behaviors, which is in line with our previous observation that pupil dilation is correlated with (reward-associated) response times to a target stimulus [12].

To our knowledge, no human study has explored the relationship between reward-anticipatory arousal regulation and depression. The goal of this study was to examine whether disruption of the physiological process of upregulating and sustaining arousal in anticipation of reward contributes to the phenotypic expression of depressive symptoms. To this end, we used simultaneous pupillometry and fMRI measurements during the above-mentioned reward anticipation task in a sample of unmedicated participants with major depression (*n* = 41) including subthreshold major depression, and 25 control participants. Our hypotheses were that depressed participants would fail to upregulate arousal as reflected in reduced pupil dilation during reward anticipation and that this would correlate with depressive symptom load. Moreover, we hypothesized that deficits in sustained arousal would originate from reduced activity in regions of the salience network—the dorsal anterior cingulate and bilateral insula.

## 2. Method

### 2.1. Subjects

One hundred sixty-one subjects were recruited as part of the BeCOME study since October 2015 (“Biological Classification of Mental Disorders” [14]) conducted at the Max Planck Institute of Psychiatry (MPIP). All subjects underwent a general medical interview and an anatomical MRI screening to rule out present/past neurological disorders or any structural brain abnormalities. In addition, all subjects participated in an intensive psychometric assessment, which involved the computer-assisted Munich version of the Composite International Diagnostic Interview (DIA-X/M-CIDI) [15], which was adapted to the BeCOME study for the assessment of current (past two weeks) symptoms of depression and anxiety. Moreover, a battery of psychometric questionnaires was included in the study, including the Beck Depression Inventory II [16]. The study protocol was in accordance with the Declaration of Helsinki and approved by the Ethical Committee of the Medical Faculty of the Ludwig-Maximilian University (350-14). All participants provided their written informed consent after the study protocol had been fully explained and were reimbursed for their participation.

Of the 161 participants who had been included in the BeCOME study until the start of our analyses (October 2018), 41 had missing pupillometry data and were excluded from all analyses. This dropout rate is fairly typical for eye-tracking within the MRI-environment, with limited amount of time to individually adjust the set-up and with some participants not strictly adhering to specific instructions. Another nine participants did have pupillometric data but had more than 15% of values missing, which we used as an exclusion criterion in our validation study [12], from which we applied exactly the same criteria and analyses in this work to prevent any post-hoc flexibility in the analyses [17]. Furthermore, an additional 10 participants had missing data of the diagnostic interview (interview aborted or technical reasons). Of the remaining 100 participants, 57 fulfilled lifetime criteria for a threshold or subthreshold major depressive episode (MDE) according to the Diagnostic and Statistical Manual for Mental Disorders, fourth edition (DSM-IV). Subthreshold depression was defined as falling short of either the symptom criterion (four instead of the mandatory five depression symptoms were reported) or the impairment criterion (symptoms were present but did not cause clinically significant impairment). Eleven of them reported a lifetime MDE that had not occurred within the past 12 months and were excluded from the main analyses. Even though medication use was a strict exclusion criterion in this study, five depressed participants were on medication (selective serotonin reuptake inhibitors, neuroleptics, or incidental benzodiazepines) and were excluded from the analyses. Comorbid disorders were no exclusion criterion for the depressed participants (see Table 1 for information on comorbid disorders).

The healthy control participants consisted of 25 individuals who did not report any lifetime diagnosis. Age range, mean, and variation as well as sex were similar in the groups (healthy control participants (*n* = 25); age range: 20–61 years, mean age = 32.1, SD = 10.3, 12 female; depressed participants (*n* = 41) range: 19–64 years, mean age = 35.9, SD = 13.4, 27 female). A majority of participants self-identified as Caucasian (78% of the depressed participants; 83% of the healthy controls). There was no evidence for no group differences regarding income and education (in years), with Bayes factors (BF_10_, see Section 2.6) of 1/3 and 1/2, respectively.

### 2.2. Psychometric Data

Depression and anhedonia severity were determined using the data from the M-CIDI. We considered the following aspects/items within the E section of the M-CIDI: (1) number of depressive symptoms in the last 2 weeks (E51A1), (2) extent to which symptoms caused impairment in daily life functioning within the last 4 weeks (E53), (3) acuteness of depression (Items E2REC and E43REC), and (4) a mean score of the anhedonia-specific items loss of pleasure (E13 and E27), loss of appetite (CE15) and loss of sexual interest (CE26). These scores were later used to explore correlations between depressive/anhedonia symptomatology and pupil readouts/BOLD activity.

### 2.3. Paradigm

**Figure 1:**
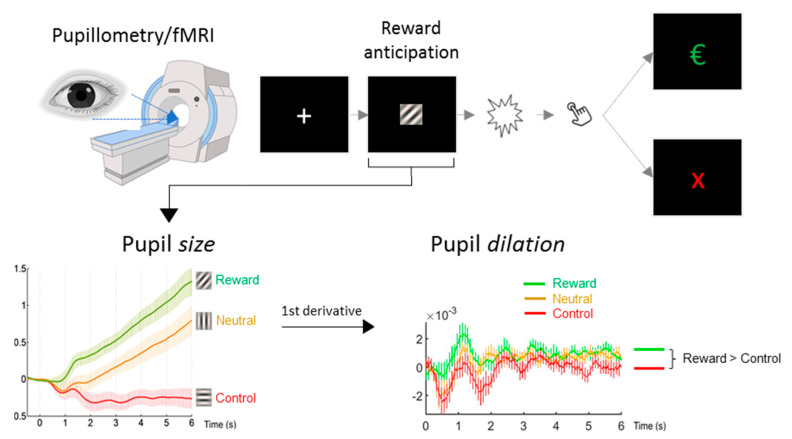
Graphic depiction of the reward anticipation task. Three isoluminant gabor patch stimuli with different stripe orientations were presented for 6 s. Pupil size was analyzed for the 6 s reward anticipation period; the increase or decrease of pupil size over time (first derivative) was used to quantify pupil dilation. Pupil dilation was averaged over the full anticipation period to obtain a robust score per stimulus and for the difference between the reward and nonresponse control stimulus. Values on the X-axis reflect time in seconds, on the Y-axes pupil size/dilation in z-transformed units. €, 1 Euro gain. X, no gain.

Subjects performed a reward anticipation task adapted from [13] inside the MR scanner, while pupil size was recorded using an MR-compatible eye tracker (Figure 1). The task utilized the same three conditions as in the original task: a potentially rewarding response condition (referred to as reward stimulus), a neutral response condition (neutral stimulus), and a control condition with no response-requirement (nonresponse control stimulus). Condition cues consisted of isoluminant gabor patch stimuli with different stripe orientations that were presented for 6 s respectively. In both response conditions, a light flash occurred after the 6 s anticipation time window, requiring a quick button press response to either obtain a monetary reward (1€) or just feedback (a green checkmark symbol). If the response was too slow, a red cross appeared instead. The response conditions were followed by a number indicating the current cumulative total win (e.g., “3€” following the third successful monetary reward trial). An adaptive algorithm ensured that participants would succeed on approximately 50% of reward trials across the session. During control trials, no flash was presented and no response was required. For a more detailed description of the experiment, see our previous publication [12]. After receiving task instructions, subjects completed an identical, two-minute training version of the task outside the scanner.

### 2.4. Pupillometry

Pupil size of the subject’s right eye was recorded at a sampling rate of 250 Hz using an MR-compatible eye tracker (EyeLink 1000 Plus, SR Research, Ottawa, Canada), which was placed at the end of the scanner bore and below the presentation monitor. Eye blinks in the pupil data were replaced via linear interpolation. Datasets with more than 15% blink/eye closure-related missing pupil values over the whole run were excluded (*n* = 9 as described above). Furthermore, to exclude trials involving poor fixation/strong shifts in eye gaze, we defined a rectangular window representing each subject’s center coordinates. Trials during which the subjects’ gaze remained outside this window for longer than 1 s and trials containing more than 50% of (blink-related) interpolated data points were discarded. Based on this criterion, M = 7.39% (SD = 11.40%) of trials had to be discarded per subject in the healthy control group, and M = 5.24% (SD = 7.93%) in the depression group.

We averaged pupil size and dilation (the first derivative of size) over the whole stimulus duration (0–6 s) to avoid multiple per-second comparisons. For group comparisons (see Section 2.6), we used mean pupil dilation for each of the three stimuli (reward, neutral, and no-response control). For correlative analyses, we again used the mean scores of the whole stimulus duration for pupil dilation, but we computed differential scores both for reward versus neutral (both conditions requiring a response) and for reward versus nonresponse control condition since the relative change was the primary variable of interest in these analyses.

To investigate the relationship between reward anticipation-related arousal and depressive symptomatology/anhedonia, we computed the correlation coefficients between pupil size and dilation and three psychometric scores of interest: the M-CIDI-based number and impact of depressive symptoms (within the last 2 weeks) and the M-CIDI-based anhedonia score. This analysis was first performed including all study participants (i.e., healthy controls and depressed participants). To rule out that potential correlations were driven by differences between healthy control and depressed participants, we repeated this analysis in the sample of depressed participants only. For correlations between pupil size and dilation and RT, we computed correlation coefficients across participant groups between pupil size and dilation and the median RTs for the two stimuli that required a response (reward and neutral stimulus).

### 2.5. fMRI

Participants were scanned in a 3 Tesla MRI Scanner (MR750, GE, Milwaukee, WI, USA) using a 32-channel head coil, covering 40 slices (AC-PC-orientation, 96 × 96 matrix, 3.0 mm slice thickness, 0.5 mm slice gap, resulting voxel size 2.5 × 2.5 × 3.5 mm^3^, echo planar imaging (EPI), TR 2.5 s, TE 30 ms, acceleration factor 2). The reward task comprised a total of 182 volumes, of which the first four volumes (i.e., 10 s) were discarded to avoid non-steady-state effects. To account for physiological artifacts and head motion-related BOLD signal changes, the following regressors were extracted and included as nuisance regressors in all further general linear model (GLM) analyses: three white matter and three cerebrospinal fluid signal regressors (a CompCorr correction), six motion regressors (extracted from the rigid body realignment step), as well as the absolute first order derivatives of these 12 regressors. This analysis was performed as in our methodological validation work [12].

The design matrices in SPM12 (https://www.fil.ion.ucl.ac.uk/spm/software/spm12/) involved one regressor per stimulus type. The following stimulus contrasts were obtained at the first level: “reward > nonresponse control stimulus” (−1 0 +1). and “reward > neutral stimulus” (0 −1 +1). To test for differences between the control and depression group, two sample *t*-tests were conducted using the first level contrast images.

As we had explicit hypotheses about regions of interest in the salience network, we extracted the individual betas (contrast reward > nonresponse control) from a 6 mm sphere around the peak voxel in the dorsal anterior cingulate cortex (dACC), left insula and right insula. From the negative contrast we extracted the betas from two main default mode network regions: the medial prefrontal cortex and posterior cingulate (see Table 2 for peak voxel coordinates). We additionally extracted the betas from the left and right ventral striatum.

### 2.6. Statistical Analyses

Group comparison analyses were Bayesian analyses as implemented in the software package JASP 0.9.2.0. (https://jasp-stats.org/). Group comparisons were performed with Bayesian repeated measures ANOVAs with group as a between-subjects factor, stimulus as a within-subjects factor, and age and sex as covariates.

In a Bayesian repeated measures ANOVA, all possible models (null model containing the subject factor, model containing the group factor alone, model containing the stimulus factor alone, model containing both, etc.) are compared and the evidence for the respective model given the data is provided as P(model|data) that sums up to 1.00. With this, one can evaluate the winning model, what the relative chances are of one model in favor of others, and what the evidence of models containing an effect or interaction compared to the evidence for the models without those effects or interactions. Additional group comparisons were Bayesian independent samples *t*-tests with standard priors (Cauchy distribution, scale: 0.7). Correlations were analyzed with Bayesian correlation analyses (stretched beta prior width = 1, which corresponds to a beta (1,1) distribution transformed to cover (−1,1) [18]. Bayes Factors (BF) are provided in favor of the alternative hypothesis compared to the null hypothesis (BF_10_), with the alternative hypothesis being undirected unless otherwise mentioned. BFs between 1 and 3 can be considered as anecdotal evidence for one hypothesis over another (1/3 to 1 is anecdotal evidence in the reverse direction), BFs between 3 and 10 (or 1/10 to 1/3) as moderate evidence, BFs between 10 and 30 (or 1/30 to 1/10) as strong evidence, BFs between 30 and 100 (or 1/100 to 1/30) as very strong evidence, and BFs more extreme than that as extreme evidence for one hypothesis over another [19].

Furthermore, in a Bayesian correlation analysis of pupil size with the total number of depressive symptoms, the observed data were modeled as samples from a multivariate Gaussian distribution [20,21]. Wide uniform priors were set for the mean of the (z-transformed) pupil dilation (−2,2) and of depressive symptoms (0,20), as well as for their standard deviations (0,2) and (0,10), respectively. The prior for the Pearson correlation was uniform as well (−1,1). The measurement error was incorporated into the model by adding one additional modeling step: the observed data were modeled as samples from true scores coming from a normal distribution with a given measurement error. The test–retest correlation was used to provide an estimate for the standard error of measurement: measurement SD × sqrt (1 − R_TEST-RETEST_). The test–retest correlation for the M-CIDI-interview was taken from published work in our population (kappa = 0.78 [15]), which would provide a conservative estimate for r. For pupillometry, we could not find published work on the test–retest reliability in a relevant cognitive-affective task, which may differ from for instance the test–retest reliability of the pupillary light reflex. We used in-house data from a pilot-study (*n* = 11) on a predictive-inference task [22], for which we split the task into four blocks on day 1 and four blocks on day 2, and observed a median test–retest correlation between the two days of 0.94 (Schneider, unpublished Master Thesis). When estimating the test–retest in our current data with the split-half reliability (trials 1–5 versus 6–10), we also observed high correlations (>0.7) for pupil dilation over the whole stimulus duration (0–6 s). We ran the analysis both with the upper and lower estimates for the test–retest reliability, with similar results. This Bayesian correlation analysis was implemented in Matlab (2018a, Natick, USA) and JAGS 4.3.0 through the Matlab trinity interface https://github.com/joachimvandekerckhove/trinity.

Finally, second level fMRI analyses were conducted within the Bayesian framework implemented in SPM12). Here a minimum threshold was selected (effect size = 0.5, logBF = 5) that has been shown to be more conservative than p_FWE_.cluster < 0.05 but more sensitive than p_FWE_.voxel < 0.05 in the frequentist approach [23]. This threshold was increased for the stimulus contrasts (effect size = 1.0, logBF = 7) to prevent single clusters from merging into one large cluster.

## 3. Results

### 3.1. Classical Group Comparison

**Figure 2:**
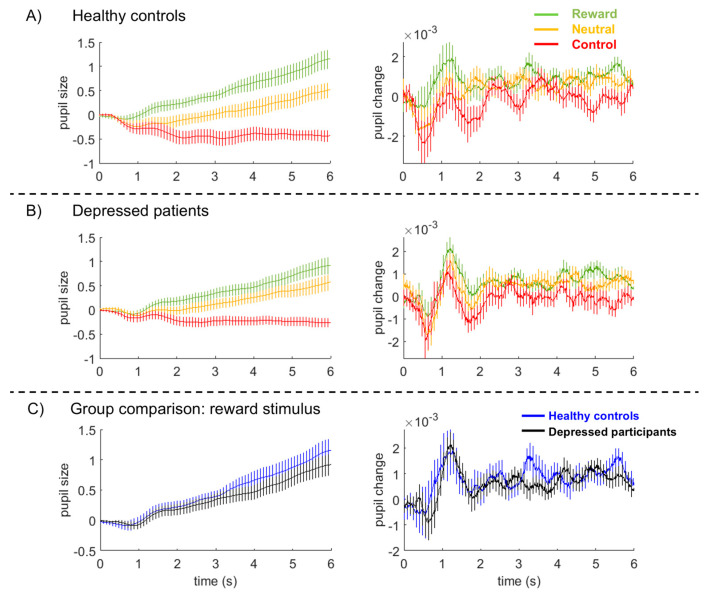
Pupil size (left panels) and pupil dilation (right panels) during presentation of the three stimuli in healthy control subjects (**A**) and depressed participants (**B**), averaged across all subjects and trials. (**C**) Average pupil size and pupil change/dilation during presentation of the reward stimulus. Pupil values are z-transformed, vertical bars represent 95% confidence intervals.

Healthy controls exhibited a strong pupil dilation in response to the reward stimulus, a moderate pupil dilation in response to the neutral stimulus, and a constriction of the pupil during the nonresponse control stimulus (Figure 2A left and right panels). Depressed participants revealed similar pupil dilation patterns to the three stimuli (Figure 2B). The Bayesian ANOVA provided no evidence for the models comprising group effects or interactions, although there was overwhelming evidence for models including the stimulus effect (P(Model given data) = 0.999 for the 12 models containing the stimulus effect versus P(Model given data) = 0.001 for the eight models without the stimulus effect).

To evaluate whether this lack of a group effect was due to the heterogeneity in acute symptomatology, we re-ran this analysis on 23 out 41 patients that reported five or more depressive symptoms within the last 2 weeks versus healthy controls. The same Bayesian repeated measures ANOVA now yielded convincing evidence for the models comprising group effects or interactions P(Model given data) = 0.887 for the four models containing the group × stimulus interaction versus 0.113 for the 16 models without this interaction, providing first indications for the relevance of acute depressive symptom load.

### 3.2. Dimensional Correlation Analyses between Depressive Symptoms and Pupil Dilation

To examine whether this relationship between depressive symptomatology and pupil dilation were more continuous, we evaluated the correlations between these variables. There was very strong evidence for negative correlations of pupil dilation during reward anticipation with both number and impact of depressive symptoms (r = −0.52 and −0.46, BF_10_ = 2363 and 284, respectively), and strong evidence for a negative correlation with anhedonia specifically (r = −0.39, BF_10_ = 23.2). Partial correlation analyses controlling for age and sex still provided strong evidence for the same negative correlations; r-values fell between −0.41 and −0.26.

**Figure 3:**
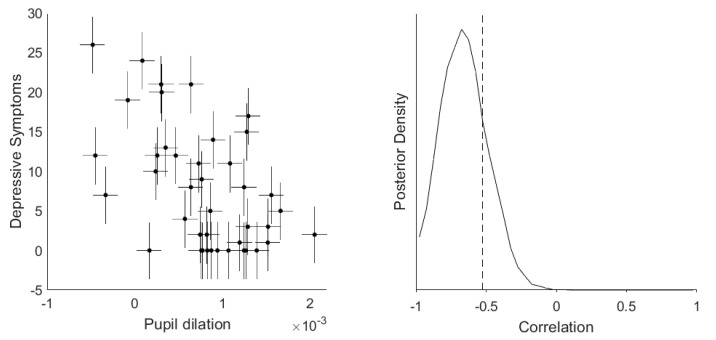
Correlation between pupil dilation and number of current symptoms with measurement uncertainty in depressed participants. Estimation of the correlation between the number of current depressive symptoms and pupil dilation to the reward stimulus with horizontal and vertical error bars representing the measurement uncertainty of the respective readouts (**left panel**). These measurement errors were incorporated into a Bayesian model that estimated the true correlations from observations sampled from a multivariate Gaussian distribution. The posterior distribution (**right panel**) provided very strong evidence that the true correlation is more negative than −0.3 (the dashed line represents the actual Pearson correlation value); the BF_10_ for r < −0.3 = 42.2).

Since these correlations could be confounded by group differences in pupil dilation and M-CIDI-scores, we re-ran them in the depressive participant group alone (Figure 3). There was again strong evidence for a negative correlation between pupil dilation and both number and impact of depressive symptoms (r = −0.53 and −0.45, BF_10_ = 80.9 and 13.2, respectively), but no longer for a correlation with anhedonia (r = −0.33, BF_10_ = 1.8) within the depressive participant group alone.

### 3.3. Correlation between Pupil Dilation and Motor Response

We found very strong evidence for a negative correlation between pupil dilation to the reward stimulus and the median response time (RT) to the same stimulus across control subjects and depressed patients, r = −0.48, BF_10_ = 581.7. Similarly, a negative correlation existed between pupil dilation to the neutral stimulus and the median RT to the same stimulus, r = −0.50, BF_10_ = 1349.1. This indicates that pupil dilation during reward anticipation tracks a process that has functional relevance.

### 3.4. FMRI Analyses

**Figure 4:**
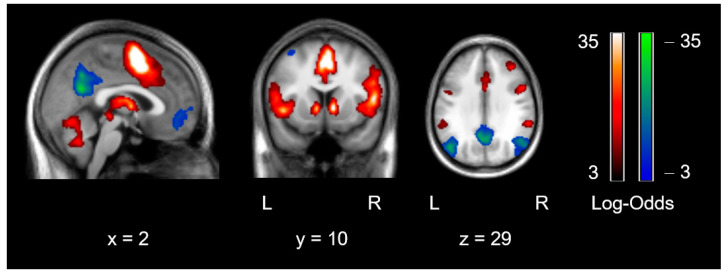
Statistical parametric maps of reward anticipation across participants and beta distributions per group. Neural correlates of a contrast representing CS+(monetary) > CS−(hot colors) and its reverse contrast (cold colors) across participants, using a threshold with an effect size of 1.0 and logBF = 7. x, y, z in Montreal Neurological Institute (MNI) space. L, left; R, right.

The stimulus contrast reward > nonresponse control (full stimulus durations) revealed very strong evidence for activity in the anterior and middle cingulate gyrus, supplementary motor area, inferior/middle occipital cortex, insula, thalamus, caudate nucleus and ventral striatum, brainstem, and cerebellum (Figure 4). The reverse contrast revealed bilateral clusters of activation in posterior cingulate gyrus, precuneus, medial frontal regions, middle occipital gyrus, angular gyrus, and temporal regions. For frequentist fMRI contrasts per group, see Supplementary Materials Figures S1 and S2.

As with our pupil dilation analysis, comparisons for the extracted ROIs between depressed participants and control participants provided weak evidence for the absence of group differences for all regions (BF_10_-values ranged from 1/4 to 1/2) except for the right insula, for which the BF_10_ = 0.8, providing neither evidence in favor of nor against group differences. We also conducted this analysis on the 23 (out of 41) patients that reported five or more depressive symptoms within the last 2 weeks versus healthy controls. The results for the right insula and the posterior cingulate cortext (PCC) now provided moderate evidence for group differences, BF_10_ = 3.1 and 4.7, respectively, whereas the BFs for the other regions remained between 1/3 and 1.

Of the three salience network regions, the correlations with the three clinical variables (symptom count, symptom impact, and anhedonia) were all weakly negative within the depressive participant group, with moderate evidence for a negative correlation between right insula and number of depressive symptoms, r = −0.42, BF_10_ = 6.4. These correlations were in the same direction as correlations between pupil dilation and clinical variables albeit weaker, which is likely due to a reduced measurement certainty of the regional beta-estimates from the fMRI analysis. When we modeled in the measurement uncertainty in the correlation analysis between dACC and impact of depressive symptoms, there was moderate evidence for a negative correlation within the depressed participants, with a BF_10_ = 3.0 for values below zero (Supplementary Materials Figure S3). Of the default mode regions, all correlations with clinical variables were weakly positive, and there was only moderate to strong evidence for a correlation between the PCC and number of depressive symptoms, r = 0.36, BF_10_ = 13.5. Moreover, the correlation of PCC with pupil dilation was also negative, r = −0.51, BF_10_ = 46.6. Controlling these correlations for pupil dilation reduced their strength to some degree (e.g., the magnitude of the correlation between the PCC and number of symptoms decreased from 0.36 to 0.27, between the right insula and number of symptoms from −0.42 to −0.27).

Finally, to examine whether we missed clusters of activity correlated to depressive symptoms, we ran post-hoc, whole-brain fMRI analyses with depressive symptomatology as a covariate of interest (see Supplementary Methods and Results). These analyses revealed one further default mode network cluster, the left angular and middle temporal gyrus [−50 −72 26], that correlated positively with both number and impact of depressive symptoms.

## 4. Discussion

We observed no robust group differences between depressed participants and healthy controls in either pupil dilation or fMRI during reward anticipation. However, we observed strong evidence for a robust negative correlation between a pupillometric measure of reward anticipatory arousal and depressive symptom load. This association existed both across healthy controls and depressive participants, as well as within the latter group only. Mirroring the pupillometric findings, task related activation in the insula and PCC also correlated with depressive symptom load in the depressive participant group. Moreover, we observed an inverse relationship between pupil dilation and response times, indicating that arousal regulation during reward anticipation has functional relevance. Taken together, these findings suggest that disturbances in the physiological process of arousal regulation contribute to the phenotypic expression of depressive states.

We interpret the observed variance in pupil dilation as directly reflecting (state-) differences in anticipatory arousal during reward anticipation. This interpretation rests on experimental work in macaque monkeys that has demonstrated a direct causal link between the neuroanatomy of salience monitoring/arousal regulation and pupil dilation during reward anticipation [10]. We could previously translate these findings to humans by use of concurrent pupillometry/fMRI, where we found that reward anticipatory pupil dilation correlated with activity in the salience network [12]. In accordance with prevailing models on the involvement of the locus coeruleus (LC) in both pupil dilation and neural gain and optimal task performance [11], we propose that after the initial reward prediction at stimulus onset, the transfer of reward prediction into action preparation might be a relevant process disturbed in participants with depressive symptoms. If LC activity is indeed lower, the neural gain signal that silences inactive regions and activates already active regions in order to prepare for the appropriate motor response, should become weaker, with reduced stimulus guided goal direction as a consequence. This hypothesis is also supported by strong evidence for a negative correlation between pupil dilation during reward anticipation and response times. Interestingly, a reduced drive to pursuit pleasurable (or necessary) activities—in German psychopathology “*Antriebsstörung*”, or “avolition” in English—is a common phenomenological feature that can be clinically observed in depressed patients. However, many self-rating questionnaires do not contain items to assess this phenomenological feature. As such, our results exemplify how objective measures of physiological processes could serve to stratify patients along dimensions that are not necessarily reflected in assessed symptoms.

Multiple other subprocesses are relevant to performing a reward task, such as reward prediction, decision-making, and reward-consumption [24]. While parallel contributions of these other subprocesses to depression pathology are likely [25,26], it is unlikely that they underlie the observed pupil dilation differences within the reward anticipatory time window, since decision making related processes and reward consumption should manifest at different time points during the task. In line with this notion, a meta-analysis of six behavioral datasets showed that depression specifically affected reward sensitivity rather than reward prediction or prediction errors [27]. Regarding reward prediction error signaling, there is evidence for between group differences in the striatum when the task contains a learning component [6,7,8]. However, this is not observed in a nonlearning task (such as the one used in our study): Rutledge, Moutoussis, Smittenaar et al. [9] showed that individuals with moderate depression exhibit reward prediction and prediction error signals in the ventral striatum that are similar to controls. Based on this, the authors concluded that depression does not affect (dopaminergic) reward prediction error signaling and that previous evidence for attenuated signaling could mirror downstream effects more closely related to aberrant behavior. The upregulation and sustainment of arousal after reward prediction is a downstream process more closely related to behavior—in our case it comprised a simple button press but in more complex tasks this could extend to approach behavior [28]. It is of note that our pupillometry data during reward anticipation do not indicate differences in general arousal levels, but specifically in the upregulation of arousal for reward-associated motor preparation. General differences in arousal would have been reflected in differences in pupil sizes at the beginning of the anticipatory phase or to all stimuli, which was not the case.

Conversely, it should be noted that differences in reward consumption between depressed patients and healthy controls have been consistently reported at the neural level (e.g., [29,30,31]). Specifically, blunted activation of the nucleus accumbens during reward consumption has been confirmed in a recent meta-analysis [32]. These findings mirror results of studies that have shown fronto-striatal circuit dysfunction in states of acute stress [33,34], potentially implicating stress related changes in reward consumption in the pathophysiology of depression [25].

Finally, the continuous relationship between acute symptom load (past two weeks) and pupil dilation during reward anticipation, independent of previous MDE diagnosis, also hints towards a state-marker characteristic of reward anticipatory arousal. More evidence regarding such characteristics will be useful for potential applications as a treatment response tracking or drug target engagement measure.

Regarding our simultaneous brain imaging data, we observed similar, but weaker associations between depressive symptom load/impact and BOLD responses within the right insula and PCC during reward anticipation. The weaker nature of these associations as compared to the correlations between pupil dilation and depressive symptom load directly, is likely due to the higher measurement uncertainty and generally lower test–retest reliability for single-subject regional fMRI beta-estimates (during reward tasks around 0.5–0.6 [35]). In line with this notion, when we modeled the measurement uncertainty in the betas extracted from the dACC (reward > nonresponse control) in a correlation analysis with clinical variables, we actually observed moderate evidence for a correlation between impact of depressive symptoms and dACC (compared to no correlation in a regular correlational analysis, which does not take into account measurement error). All in all, these results indicate that salience network regions correlate negatively with depressive symptom load—paralleling pupil dilation or possibly partially mediated by pupil dilation, as indicated by the partial correlation analyses. This is interesting as it lends further evidence to the notion that fMRI measures of the salience network—such as resting state connectivity metrics—reflect a disease-relevant physiological process. This is in line with results from a recent meta-analysis that implicated disruption in the salience network in depression and trans-diagnostically across psychiatric disorders [36]. In contrast, the PCC—as part of the default mode network that deactivates in our task—shows the opposite pattern, which represents a failure to reduce default mode network activity during reward anticipation. This is in accordance with previous work that suggests differences in task-related default mode network activity in depressed participants [37,38].

A compelling feature of pupillometry is that it provides indirect access to the central monoaminergic regulatory systems that are implicated in a wide variety of cognitive and emotional functions. This has been utilized in the past to characterize depressed populations regarding differences in the physiological response to various tasks, such as working memory [39], cognitive control [40,41], and emotional processing [42,43,44,45,46,47]. Further, differences in the more direct autonomic regulation of the pupil have been studied in depressed populations, such as pupillary unrest as a measure of vigilance and spontaneous arousal fluctuations [48], or the pupillary light reflex [49,50,51,52]. Our works extends this body of work by highlighting the physiological process of arousal upregulation during reward anticipation in the pathophysiology of depression.

Although numerous studies provide strong evidence for a close relationship between pupil dilation and noradrenergic activity in rodents, primates, and humans [11,53,54,55], other neuromodulatory systems like the cholinergic and serotonergic system are known to influence pupil dilation as well [56]. For example, Reimer, McGinley, Liu, Rodenkirch, Wang, McCormick, and Tolias [55] found that in mice, spontaneous pupil fluctuations track both changes in noradrenergic and cholinergic activity. Interestingly, they observed that fast pupil dilations typically occurring during rest and at the beginning of walking on a treadmill were closely linked to phasic noradrenergic activity, whereas longer-lasting dilations observed during continuous locomotion were accompanied by sustained cholinergic activity. Their finding seems to fit our interpretation of a noradrenaline-mediated upregulation of arousal that facilitates subsequent (goal-directed) behavior, however, we cannot rule out that pupil dilations observed in the present study also involve a cholinergic component. Another neurotransmitter that has been associated with changes in pupil size is serotonin: for example, serotonergic agonists like lysergic acid diethylamide (LSD) have been shown to cause a dilation of the pupil [57], which has been suggested to result from interactions with LC neurons and subsequent release of noradrenaline [56,58,59]. Since patients included in the present study were unmedicated and had a similar baseline pupil size as healthy control subjects, we can rule out that the observed group differences in pupil dilation might reflect differences in antidepressant medication. However, more studies will be needed to precisely disentangle noradrenergic, cholinergic, and serotonergic influences on pupil size.

One further limitation of this study is the limited sample size of healthy controls. This was in part due to the rigorous phenotyping of all subjects that included an extensive M-CIDI assessment, whereby a substantial portion of subjects that were recruited as healthy controls turned out to fulfill psychiatric diagnoses. However, since we were primarily interested in continuous associations between differences in reward anticipatory arousal and depression symptom load, the role of healthy subjects was to span the variance in our study population and not to increase power for group comparisons. Therefore, our data also highlight the limitations of classical patient–control comparisons and instead argue for more dimensional approaches [60,61].

## 5. Conclusions

We showed robust pupillometric correlational findings as well as neural measures of salience network functioning that converge to evidence for a disruption of reward-anticipatory arousal regulation in depressed states. To our knowledge, this is the first time that this neuroanatomically well delineated process [10] has been implicated in the pathophysiology of depression. The ability to track this process directly in individual patients via pupil responses makes this a promising marker for a physiologically informed patient stratification and could inform novel interventions that target this specific process.

## Figures and Tables

**Table 1:** Frequency of comorbid DSM-IV 12-month and lifetime diagnoses in the depressed participants (*n* = 41).

	12-Month		Lifetime	
DSM-IV Diagnoses	*n*	%	*n*	%
Any anxiety disorder	22	53.6	21	51.2
Panic disorder	5	12.2	3	7.3
Specific phobia	17	41.5	5	12.2
Generalized Anxiety Disorder	0	0	11	26.8
Posttraumatic Stress Disorder	2	4.9	4	9.8
Any substance use disorder	3	7.3	10	24.4
Any comorbid diagnosis	9	22.0	20	48.8
Just one comorbid diagnosis	6	14.6	10	24.4
Two comorbid diagnosis	3	7.3	6	14.6
more than two comorbid diagnoses	0	0	4	9.8
Comorbid with just an anxiety disorder	0	0	3	7.3
Comorbid with an anxiety and a substance use disorder	0	0	3	7.3

Substance use/dependence disorders (nicotine, cannabis, and/or alcohol) all had a recency of >12 months, with the exception of *n* = 2 for nicotine dependence that had a recency >7 months, and *n* = 1 for nicotine dependence with a recency >1 month, and *n* = 1 with current nicotine dependence. DSM-IV, Diagnostic and Statistical Manual for Mental Disorders, fourth edition.

**Table 2:** Peak voxel coordinates for region-of-interest analyses.

Region Name	MNI Coordinates	Cluster Extent	Peak Log Odds
Contrast [reward > nonresponse control]			
Dorsal anterior cingulate	[8 14 40]	3013	36.0
Insula (L)	[−36 16 −6]	2724	36.0
Insula (R)	[36 20 −10]	2572	36.0
Ventral striatum (L)	[−8 8 0]	1337	36.0
Ventral striatum (R)	[10 6 −2]	„	„
Contrast [reward < nonresponse control]			
Posterior cingulate	[10 −56 20]	1848	34.7
Medial prefrontal cortex	[−6 56 0]	786	22.1

The contrast from which these clusters were generated is depicted in Section 3.4. It had an effect size of 1.0 as the threshold, together with logBF = 7 and cluster extent = 50.

## Supplementary Materials

The following are available online at https://www.mdpi.com/2076-3425/10/12/906/s1, Figure S1: Neural correlates of a contrast representing the reward > control stimulus., Figure S2: Neural correlates of a contrast representing the reward > neutral stimulus., Figure S3: Correlation between beta-values of the dACC and impact of depressive symptoms with measurement uncertainty.
