# Supplementary material for: Pupil Dilation during Reward Anticipation Is Correlated to Depressive Symptom Load in Patients with Major Depressive Disorder
Source: Brain Sci. 2020 Nov 25;10(12):906. doi: 10.3390/brainsci10120906 (PMC7760331; doi:10.3390/brainsci10120906)
Supplement: Supplementary file 1 [file brainsci-10-00906-s001.pdf]

brain sciences

MDPI

Supplemental Material

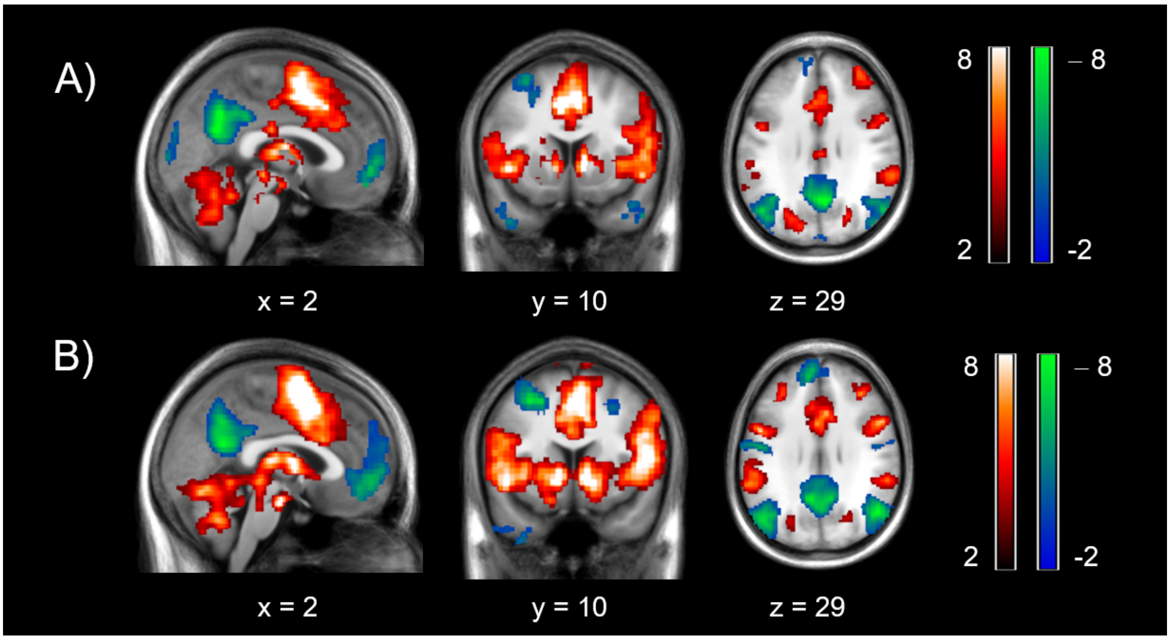

Suppl. Fig. 1. Neural correlates of a contrast representing the reward > control stimulus. The contrast representing the reward > control stimulus (hot colors) and its reverse contrast (cold colors) was computed for A) healthy controls (N = 25) and B) depressed participants (N = 41). Statistical maps were collected at uncorrected $p < 0.001$ and clusters were thresholded at a cluster-wise $p_{FWE} < .05$ (resulting in an effective cluster extension threshold of $k > 130$ for display purposes).

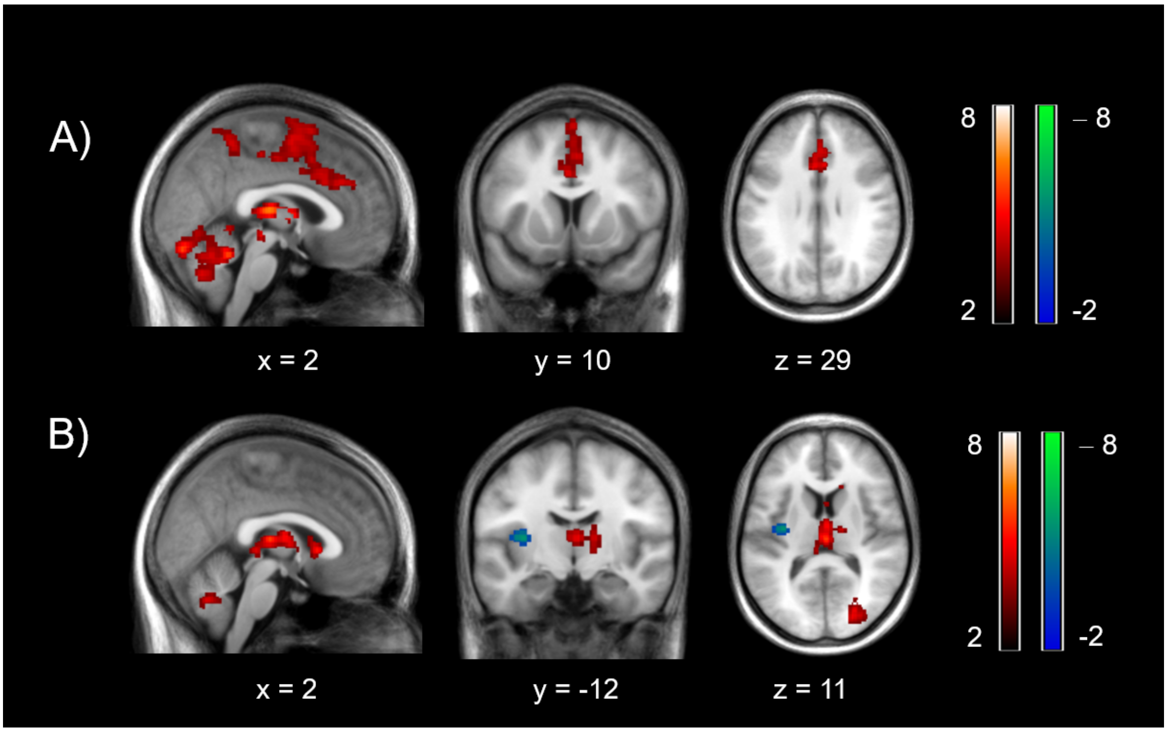

Suppl. Fig. 2. Neural correlates of a contrast representing the reward > neutral stimulus. The contrast representing the reward > neutral stimulus (hot colors) and its reverse contrast (cold colors) was computed for A) healthy controls (N = 25) and B) depressed participants (N = 41). Statistical maps were collected at uncorrected $p < .001$ and clusters were thresholded at a cluster-wise $p_{FWE} < .05$ (resulting in an effective cluster extension threshold of $k > 100$ for display purposes).

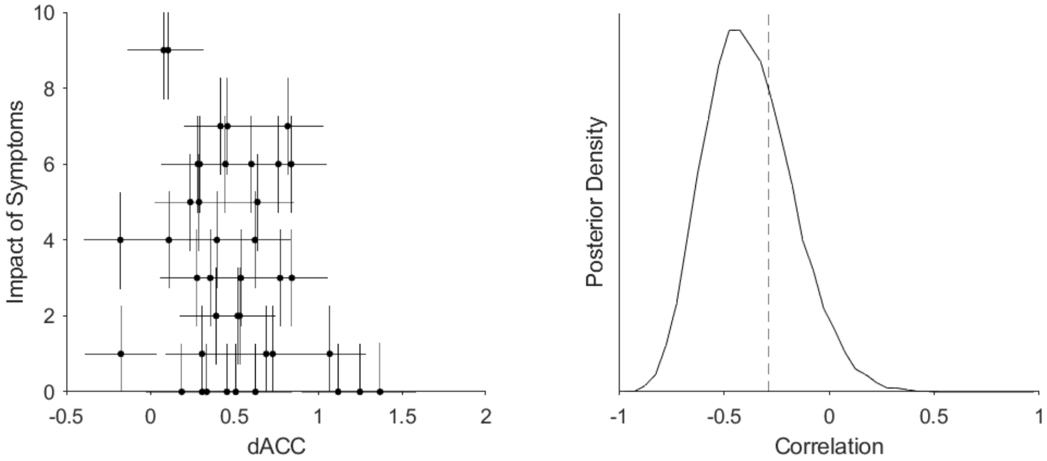

Suppl. Fig. 3. Correlation between beta-values of the dACC and impact of depressive symptoms with measurement uncertainty. Estimation of the correlation between the impact of depressive symptoms and dACC beta-values from the contrast reward > control stimulus, with horizontal and vertical error bars representing the measurement uncertainty of the respective readouts (left panel). The measurement error was estimated for the M-CIDI-interview by the test-retest reliability (Kappa ~ 0.78 in our population, see [15]), and for dACC beta-values we took the value of 0.6 estimated from the test retest reliability of a monetary incentive delay task [35]. These measurement errors were again incorporated into the same Bayesian model that was used to generate Figure 3; in this case it provided moderate evidence that the correlation between dACC and impact of symptoms was smaller than 0, $BF_{10} = 3.0$.

Supplementary Methods and Results

To examine whether we missed correlations between depressive symptomatology and reward-related activity in other brain regions, we ran multiple whole-brain fMRI analyses. We started with the contrasts from which we extracted the regional values (contrast reward > non-response control) and entered the values of depression symptoms and pupillary responses as covariates in the second level analyses. This was performed with a mask of the task-effect that was thresholded at uncorrected $p < 0.005$ (F-contrast). We conducted the following analyses:

1. The number of depressive symptoms, age and gender as covariates; both groups;
2. The impact of depressive symptoms, age and gender as covariates; both groups;
3. The differential pupil response, age and gender as covariates; both groups;
4. The number of depressive symptoms, age and gender as covariates; depressive group only (N = 41);
5. The impact of depressive symptoms, age and gender as covariates; depressive group only (N = 41);
6. The differential pupil response, age and gender as covariates; depressive group only (N = 41).

Statistical maps were collected at uncorrected $p < .001$ and clusters were thresholded at a cluster-wise $p_{FWE} < .05$. We only observed one cluster of activity in the left angular gyrus / middle temporal gyrus in analyses 4 ($p_{cluster} = 0.007$, k = 207, t = 4.80, MNI coordinates of the peak voxel [-50 -72 26]) and 5 ($p_{cluster} = 0.006$, k = 215, t = 4.46, MNI coordinates of the peak voxel [-54 -72 16]), reflecting a positive correlation with the number and impact of depressive symptoms, respectively.
